# Resilience as the Ability to Maintain Well-Being: An Allostatic Active Inference Model

**DOI:** 10.3390/jintelligence11080158

**Published:** 2023-08-07

**Authors:** Christian E. Waugh, Anthony W. Sali

**Affiliations:** Department of Psychology, Wake Forest University, Winston-Salem, NC 27109, USA; saliaw@wfu.edu

**Keywords:** resilience, well-being, allostasis, active inference

## Abstract

Resilience is often characterized as the outcome of well-being maintenance despite threats to that well-being. We suggest that resilience can also be characterized as an emotional-intelligence-related ability to obtain this outcome. We formulate an allostatic active inference model that outlines the primary tools of this resilience ability as monitoring well-being, maintaining stable well-being beliefs while updating situational beliefs and flexibly prioritizing actions that are expected to lead to well-being maintenance or gathering the information needed to discern what those actions could be. This model helps to explain the role of positive emotions in resilience as well as how people high in resilience ability use regulatory flexibility in the service of maintaining well-being and provides a starting point for assessing resilience as an ability.

## 1. Introduction

The author CW has a friend (we will call him “Bob”) who is really gifted in carpentry—he makes beautiful furniture. One day, CW asked him about his secret and he responded that building furniture requires the right tools. So, over the course of several years, CW spent too much money buying cool tools. Alas, CW’s furniture never quite compared to Bob’s. Bob further explained that one also needs the ability to use those tools effectively, so CW watched YouTube videos and practiced, and although he was able to cut wood and attach it to other pieces of wood better, his furniture was still not quite as good as Bob’s. Finally, it dawned on CW that Bob’s skill at using those tools effectively went beyond accomplishing the subgoals of cutting and drilling to serve a well-visualized end product or outcome that he was trying to achieve. So, good carpentry is about having the right tools and having the ability to use those tools to accomplish a well-visualized outcome.

It is inarguable that crafting a beautiful piece of furniture involves one’s abilities, but what about crafting a resilient life? Surprisingly, this is a little less clear because investigators have been inconsistent with conceptualizing resilience as the properties/abilities of the person (Block and Kremen 1996) or as the outcome of a process (Masten 2001). An example of the former is Block’s characterization of resilience as “the dynamic capacity of an individual to modify a characteristic level of ego-control, in either direction, as a function of the demand characteristics of the environmental context… (Block and Kremen 1996, p. 351).” For resilience researchers like Block, resilience is characterized as the ability of the person to adapt to their environment. On the other hand, an example of the latter conceptualization of resilience as a process comes from stress researchers who define resilience as a relative stability in functioning from before to after some adverse event (e.g., Bonanno et al. 2002; Masten 2001). Returning to our furniture metaphor, resilience has been treated both as the ability to use tools to make the furniture and as the furniture itself.

One reason for this confusion is that resilience can be appropriately conceptualized as both an ability and an outcome in the same way that intelligence can. An intelligent person, like Albert Einstein, has the ability to create intelligent outcomes, like the theory of relativity. Similarly, a resilient person, like John McCain, has the ability to create resilient outcomes, like successfully surviving and thriving after being held as a prisoner of war for over 5 years. However, conceptualizing resilience as both the ability and the outcome yields circular reasoning that X ability necessarily leads to X outcomes and X outcomes are necessarily produced by X ability. To avoid this circular reasoning, we must recharacterize either the resilience ability or resilient outcome. There are many ways to accomplish this, but in the spirit of this special issue on emotional intelligence, we will focus on resilience as an ability and recharacterize resilient outcomes.

## 2. Resilience as an Ability

To characterize resilience as an ability, we begin with research on emotional intelligence (EI), which provides nice guidelines for understanding what constitutes emotion-related abilities (Mayer et al. 2016). According to EI, an “ability” is the capacity to enact the behaviors required to accomplish some goal and/or solve some problem. For example, the four-branch model of EI proposes a hierarchical organization of problem-solving areas. From the most computationally basic to the most complex, these consist of perceiving emotions, facilitating thought using emotions, understanding emotions and managing emotions (Mayer et al. 2016). Furthermore, for each of these problem-solving areas, there exists a collection of abilities that are associated with high emotional intelligence (see Table 1 in Mayer et al. 2016). If EI is a collection of abilities, as the four-branch model proposes, then maximum performance measures should provide a better measure of EI ability than individuals’ self-reported EI. Consistent with this logic, scores from the Mayer–Salovey–Caruso Emotional Intelligence Test (MSCEIT), a well-validated performance measure of EI, but not self-ratings from the Self-Rated Emotional Intelligence Scale, are associated with measures of social competence and the two measures of EI are only weakly correlated with each other (Brackett et al. 2006). Therefore, the conceptualization of resilience as an ability may be aided by the development of new performance measures in addition to the existing self-report measures (such as trait scales: Block and Kremen 1996).

Some researchers have suggested that EI is a core ability underlying resilience (Salovey et al. 1999), which prompts the question of whether we need to define resilience as a separate ability at all. Indeed, the skills associated with EI, such as managing and understanding emotions, can help people cope successfully (Salovey et al. 1999). For example, self-reported EI has been shown to predict decreased stress responses to life stressors (Armstrong et al. 2011) and EI is measured as an ability correlated with a better response to a laboratory stressor (Schneider et al. 2013). However, the link between EI and resilience does not seem to be as strong as to suggest that they might be the same ability. First, EI is not universally related to positive outcomes after stress. One study showed that EI ability did not predict social stress responses (Matthews et al. 2006). Second, the link between EI and good stress responding appears to be moderated by other vulnerabilities (Davis and Nichols 2016). For example, having high emotion perception (a component of EI ability) led to a stronger relationship between hassles and depression (Ciarrochi et al. 2002) and high EI predicted increased cortisol reactivity and prolonged stress recovery for those high in basal testosterone (Bechtoldt and Schneider 2016). Summing up these mixed findings, Zeidner et al. 2006 suggest that successful adaptation and resilience is a multivariate outcome and that EI may be just one component.

We feel justified then in characterizing resilience as its own ability that features EI as one of the skills that helps enable people to be resilient. Further, it is useful to use the ability approach of EI to craft this characterization of resilience ability. One tenet of this approach is that because having an ability enables the enactment of behaviors, this ability exists outside of and must precede the enactment of those behaviors. In other words, to accomplish an emotionally intelligent behavior like understanding that one’s partner is feeling frustrated, one must first be emotionally intelligent and able to understand others’ emotions. If resilience can be characterized as an ability, we must accept that people have “resilience-ability” before they accomplish resilient outcomes. Consistent with this idea, researchers who have treated resilience as a relatively stable trait (e.g., Block and Kremen 1996) found that reporting higher trait resilience on a scale at one time point predicted reduced depression after a national tragedy like the terrorist attacks on the US on 9/11 (Fredrickson et al. 2003) and successful stress recovery in the lab (Tugade and Fredrickson 2004). Importantly, characterizing resilience as a trait-like ability suggests that it might be relatively stable like other traits, but, like other abilities, it can be developed and learned in those who may not yet have resilience ability (Waugh and Koster 2015). For example, stress inoculation research suggests that resilience can be developed in childhood through experiencing mild stressors in a supportive environment (Seery et al. 2010). Resilience can also be developed in adulthood (Waugh and Koster 2015); researchers have found that the ability to flexibly express emotions subsequently predicts decreased distress during the first two years of college (Bonanno et al. 2004) and emotional expressivity is an ability that can be improved (Giese-Davis et al. 2002).

The second tenet of this ability approach to resilience is that an ability is the capacity to enact certain behaviors to solve problems and accomplish goals. As noted before, it is circular reasoning, and therefore unhelpful, to say that the ability to be resilient is related to the capacity to have resilient outcomes. So, we must more precisely define what constitutes a “resilient outcome” to characterize the goals that a high resilience ability can achieve. We start with the typical way of defining resilient outcomes. In common parlance, resilience is thought of as the ability to “bounce back” from adverse events (e.g., https://positivepsychology.com/what-is-resilience/, accessed on 7 March 2023). Indeed, the word resilience is originally a metallurgy term that characterizes the ability of a metal to “bounce back” to its original shape after being bent. Similarly, Bonanno and colleagues have defined resilience trajectories after trauma as those in which people exhibit no significant change to their pretrauma levels of functioning (Bonanno 2004).

Furthermore, resilience is associated with and characterized by having high well-being and good functioning in spite of threats to that functioning (Masten 2001). One of the original longitudinal studies on resilience in the children of Kauai is powerful because it describes how well they are able to function and exhibit high well-being despite their desolate living conditions (Werner and Smith 1992). Bonanno and colleagues differentiated those who had stable patterns of good functioning (resilient) from those who had stable patterns of poor functioning (chronic dysfunction) from pre- to posttrauma (Bonanno 2004).

Next, “high levels of functioning” feels a little vague/broad. Technically, if we leave the definition of resilience broad, then the maintenance of any type of good functioning could count. For example, one might be physically resilient if one is able to maintain the functioning of one’s overall health after an illness. In this article, we are focusing on resilience in the realm of mental health, so high levels of functioning should reflect that (although, see conceptualizations that draw parallels between physical and mental resilience: Davydov et al. 2010). Well-being is a good candidate given that well-being reflects high levels of mental health functioning and is a common outcome measured in studies on resilience (e.g., Fredrickson et al. 2003). In the next section, we more fully unpack this formulation, but in the meantime, our characterization of resilience ability is “the ability to maintain high well-being in spite of threats to that well-being.”

Returning to EI, it is clear that EI predicts well-being, so is it that different from resilience ability as defined above? We contend that resilience is a higher-order ability that includes EI. Whereas EI ability is made up of constituent lower-order skills (emotion management, emotional perception, etc.) that tend to predict high well-being (Sánchez-Álvarez et al. 2016), resilience is the ability to maintain well-being by using whatever lower-order skills are necessary (including, but not exclusively limited to, EI; Zeidner et al. 2006). Because EI and resilience ability are hierarchically situated, EI skills form part of the suite of resilience skills.

In the next sections, we flesh out our characterization of resilience as an ability. We also provide some preliminary suggestions on ways to assess resilience ability. We must note that, because we are putting forth a novel characterization of resilience ability, most of these assessments have not been fully validated as tests of this new characterization and some have not been tested at all. However, we hope that these ideas may be fruitful for future investigations into resilience ability.

## 3. Well-Being Maintenance

Well-being has been traditionally characterized in terms of hedonic/subjective well-being and eudaimonic well-being. Subjective well-being consists of life satisfaction, a cognitive component relating to one’s overall evaluation of how one’s life is going, as well as overall high levels of positive emotion and low levels of negative emotion (Diener et al. 1999). Alternatively, eudaimonic well-being consists of meaning-related processes like autonomy and mastery (Ryan and Deci 2001) as well as self-acceptance and purpose in life (Ryff and Keyes 1995).

Hedonic and eudaimonic well-being may be more related to each other than was historically theorized (Kashdan et al. 2008) and, although a full treatment of this debate is beyond the scope of this article, we have found it useful to focus on “positive appraisal” as a mechanism common to both types of well-being. Specifically, we (and others; Caprara et al. 2010) have suggested that well-being can be construed in terms of positive appraisals/evaluations of one’s overall life (life satisfaction) and circumstances (positive emotions), which map onto subjective well-being, as well as positive appraisals/evaluations of one’s goals (purpose/meaning) and relationships (belonging), which map onto eudaimonic well-being (Waugh 2023).

One of the, perhaps, surprising conclusions that resilience researchers have drawn in the last couple of decades is that resilience seems to be quite common (Bonanno 2004; Masten 2001), and they have come to this conclusion because they have found that the maintenance of well-being is quite common (Diener and Diener 1996). Numerous studies have found that, on average, well-being is quite stable over time (Eid and Diener 2004), even when accounting for potentially life-changing events (Costa et al. 1987). This stability has also caused investigators to question the prevalence of actual posttraumatic growth—a reliable increase in well-being after a severe stressor or traumatic event—suggesting instead that most of this phenomenon as currently measured is due to people’s beliefs that they have grown and less to actual lasting changes in their well-being (Jayawickreme and Infurna 2021).

Well-being tends to be stable because it tends to be more related to people’s stable characteristics and traits than to temporary states (Eid and Diener 2004; Hudson et al. 2020). Well-being is quite strongly associated with lower neuroticism and higher extraversion (Steel et al. 2008), higher optimism (Scheier and Carver 1992) and has a strong hereditary component (Keyes et al. 2010). When making judgments about how life is going overall, people tend to use chronically accessible information about themselves (e.g., identity, life goals) that are commensurate with their personality traits rather than use more temporarily accessible information (e.g., the weather) that is more variable (Robinson and Clore 2002; Schimmack and Oishi 2005).

It is important to distinguish well-being as a global, relatively stable evaluation/appraisal of one’s life from moods and emotions, which are temporary responses to life events. When assessed as a global evaluation, well-being has been shown to be more stable than “occasion-specific” states (Eid and Diener 2004) such as moods (Gadermann and Zumbo 2007; Hudson et al. 2020) or well-being assessments that are anchored to the moment (Busseri and Newman 2022; Sonnentag 2015). Even when assessing positive and negative affect as a component of subjective well-being (Diener et al. 1999), there is a stronger relationship between global life satisfaction and measures that assess positive affect over the course of a considerable amount of time (e.g., two weeks) than in the moment (Hudson et al. 2022).

Although well-being seems to be stable on average, there are important individual differences in that stability. First, some major life events challenge people’s well-being stability more than others. For example, although people tend to return to a well-being set-point (their prior levels of well-being) after marriage, people’s well-being tends to decrease and not return to a set-point after severe disability (Lucas 2007). Furthermore, when examining within-person well-being trajectories from before to after major life events, there appears to be substantial between-person variability (Lucas 2007).

We argue that individual differences in resilience ability produce these individual differences in well-being stability. Lower variability in well-being is associated with higher conscientiousness, perceived support and lower negative mood (Gadermann and Zumbo 2007). Also, those higher in well-being before a major life event tend to exhibit more stable well-being after that event (Lucas 2007). We contend, therefore, that the degree to which these characteristics predict well-being stability, especially in times of adversity, are characteristics of resilience ability. For example, conscientiousness is associated with the ability to solve problems effectively, which, in turn, is crucial to the ability to maintain well-being during adversity (Campbell-Sills et al. 2006).

### Assessing Well-Being Maintenance

Researchers in EI have made compelling cases for the value of performance-based metrics of EI that do not rely on someone just reporting that they think that they have EI (Mayer et al. 2016). It would therefore be beneficial if researchers were able to construct similar performance-based metrics to assess the ability to maintain well-being in the face of well-being threats. However, measuring well-being maintenance with performance-based metrics would be difficult. Because EI is a form of intelligence, the performance metrics involve the tester administering problems that the test-taker solves (e.g., identifying an emotional expression or describing when the most appropriate time would be to regulate an emotion). For resilience, this is difficult because the problem being administered would have to be “threats to one’s well-being” and there are significant ethical and moral problems with manipulating such a threat on a scale that could genuinely impact people’s overall well-being (and not just their momentary mood). Therefore, for assessing the ability to maintain well-being despite threats to that well-being, we must be satisfied with self-reports of well-being in the context of naturally occurring life events, which we describe below. That said, we propose in later sections how to potentially assess the skills/tools that underlie this well-being maintenance ability using more traditional performance metrics.

Measuring the maintenance of high well-being could be as simple as assessing someone’s (in)variance of well-being over some particular time frame (Lucas 2007). Common metrics for measuring (in)variance are standard deviations and root-mean-successive-squared difference (Dejonckheere et al. 2019). Importantly, however, this suggests that just measuring well-being at one time point is not sufficient to demonstrate resilience ability and that to get a good metric of variability would likely require many samples of well-being (Lucas 2007). An important goal of future research would be to determine what the timing of those well-being samples would be to capture their stability and distinguish them from more momentary contextual emotions and moods. Also, this suggests that measuring well-being longitudinally, whereby the primary dependent variable is well-being at one time point, controlling for well-being at prior time points does not reflect resilience ability per se. Indeed, if an individual has high resilience ability, then they should show stability in their well-being over time, not necessarily improvements that are unrelated to prior well-being states. Positivity interventions, those designed to improve well-being (Parks and Biswas-Diener 2013), are therefore not resilience interventions unless they can also show that they lead to greater stability in people’s well-being.

This stability of well-being is only valuable in so far as it is paired with having high well-being, otherwise, we might capture chronic and stable distress (Bonanno 2004). Thus, measurements of resilience ability must account for the mean level of well-being in a way that also factors in its (in)variance. One avenue for future research is identifying the type of relationship between (in)variance and mean well-being (e.g., ratio? combinatorial?) as well as the weight put on each metric (e.g., equal weight? greater weight towards the mean?) that accurately captures resilience ability.

**Figure 1:**
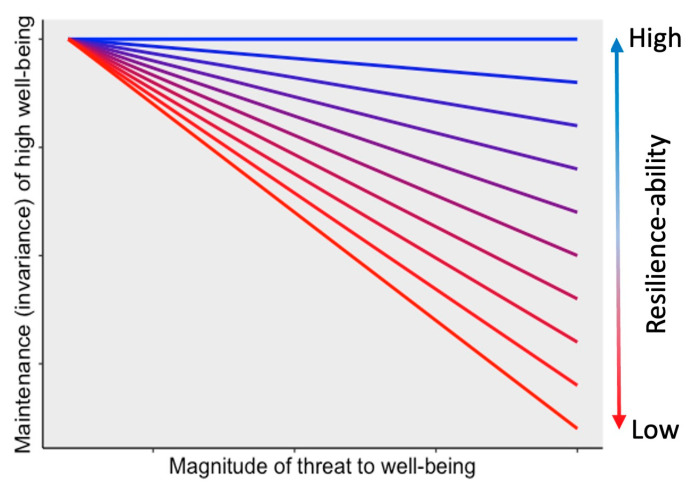
The proposed relationship between maintenance of high well-being and magnitude of the threat to well-being as a function of resilience ability. Note that this graph only portrays the maintenance of high well-being, because low well-being would just represent low resilience ability regardless of the magnitude of threat to well-being.

Lastly, resilience ability measurements need to assess threats to well-being. Often, these threats are assessed in one of several ways: a specific, identifiable adverse event (e.g., mass shooting), a culmination of life experiences (e.g., poverty) and/or a self-report assessment of life stressors (Holmes and Rahe 1967). Measuring life stress is a complicated process and scientists should aim to assess it as independently as possible from psychological responses to that stress (Monroe and Harkness 2005), through structured interviews (Monroe and Harkness 2005) and/or life event checklists that are standardized to established norms (e.g., Holmes and Rahe 1967), while taking into account cultural differences in those norms (Troy et al. 2023). Once life stress is measured, then one may be able to calculate within-person slopes that represent the relationship between stress and well-being maintenance over time as an indicator of resilience ability (Figure 1; Mroczek and Almeida 2004).

## 4. Resilience Tools to Maintain Well-Being: An Allostatic Active Inference Model

We have, so far, posited that resilience ability is the ability to maintain well-being in spite of threats to that well-being. To exercise this ability would require people to use various tools and strategies. Therefore, maintenance of well-being is not just accomplished via homeostasis—maintaining a set-point through local mechanisms—but rather through allostasis—maintaining stability through change (McEwen 1998). Instead of thinking about stress, or “threats to well-being”, as negative, allostasis offers an alternative model in which stress induces psychological and physiological responses, which can be adaptive because they provide the energy and altered cellular functioning needed to address the demands elicited by the stressor. For example, one’s heart rate rises during physical exertion to provide blood and oxygen to the muscles and organs needed to successfully do that exercise.

Allostasis is an active system in which the brain strategically deploys energy to prepare responses to predicted stressors. This active system requires the ability to enact those allostatic strategies, and it has been proposed that resilience is essentially this ability (Karatsoreos and McEwen 2011). Indeed, if we reframe allostasis as “maintaining well-being stability through change” then we have a framing that is quite close to our definition of resilience ability. Thus, the principles of allostasis can inform the tools and strategies underlying resilience ability.

One principle of allostasis is that it is a predictive system (Sterling 2012). Organisms monitor potential demands from the environment and if a demand is expected, they change their physiological and psychological response systems to prepare for it. Back to our heart rate example, this predictive system is why our heart rate increases in anticipation of psychological and physical stressors (Waugh et al. 2010).

Active inference is a framework that can speak to how this monitoring and predictive responding occurs (Barrett 2017; Friston et al. 2017; Smith et al. 2022). At the heart of active inference is the principle that people are always making predictions of the future state of the world and monitoring how their actual observations of the state of the world compare with their predictions. Furthermore, when choosing among several possible actions, individuals can simulate the states of the world that are likely to follow each possible action. This internal probabilistic model allows them to select the action that will lead to a preferred outcome. Following action selection, the individual must update their model of the world in a fashion that minimizes the difference between their predicted and observed experiences, referred to as the prediction error.

As an example, imagine that a ball player is at bat with a full count of three balls and two strikes. The batter knows that they must select an action (to swing or to not swing) before they have full sensory information and must therefore base their decision on predictions about the upcoming state of the world. As the pitcher begins to release the pitch, the batter notices the arm angle of the pitcher and does some quick calculations about where the pitch will be. In this case, they decide to refrain from swinging, predicting that the pitch will land outside of the strike zone. If the pitcher does in fact throw outside of the strike zone, the difference between the batter’s predicted outcome and the observed outcome will be small, requiring very little updating of the internal model. However, if the pitcher throws a strike and the batter strikes out, there is a larger discrepancy between the predicted and observed outcomes. The batter must then update their predictive model so that when they are next in this situation, they will select a more optimal action. In the context of our current discussion, resilience ability reflects the capacity to monitor well-being, predict potential threats to well-being and enact the actions needed to minimize the extent to which these changes occur.

According to the active inference framework, selecting the best course of action requires minimizing two forms of “free energy” at different stages in the process. When there are multiple sets of updated beliefs that could minimize prediction error, minimizing variational free energy (VFE) involves balancing the minimization of prediction errors while seeking parsimony, or the least drastic belief change possible. In the batting example, the batter’s prediction was wrong, but they need to balance updating that prediction given their understanding of pitching mechanics with other extraneous variables that could have impacted the pitch (e.g., wind or a bad call by the umpire).

**Figure 2:**
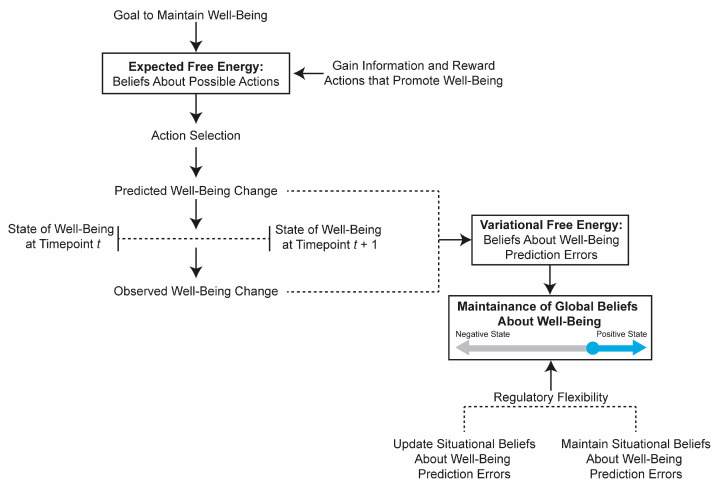
An allostatic active interference model of well-being. To achieve the goal of high well-being, individuals select actions that promote positive well-being changes and information gain, known as minimizing expected free energy. During this action selection, an internal model estimates the likelihood of well-being change outcomes, allowing the individual to make predictions. After performing the action, individuals observe how the actions impacted their well-being and must update their predictive model to minimize the degree to which the model predicts the wrong outcome in the future. This prediction error along with a valuation of parsimony is known as the variational free energy (VFE). Individuals with high resilience ability maintain stably positive global beliefs about well-being in the face of threats and flexibly regulate when to update and when to maintain situational beliefs about well-being.

In terms of well-being maintenance, we suggest that this trade-off between belief stability and updating is hierarchical (Figure 2). At the top level are overall beliefs about one’s well-being—those positive appraisals of one’s past, present and especially one’s future. At this top level, resilience ability should be associated with minimizing VFE mostly by maintaining stable beliefs about one’s well-being, which is essentially the argument we made in the prior section. Constantly updating one’s belief about well-being due to minor prediction errors would cause instability in well-being and poor resilient functioning. But what about major well-being prediction errors (i.e., things are worse off than people expected them to be; not necessarily misprediction of the event’s occurrence) like divorce, death of a loved one, etc.? We know that those major prediction errors can and do affect people’s levels of well-being (Lucas 2007), but not for those at higher levels of resilience ability (Bonanno et al. 2002). There are a couple of possible explanations. First, it could be that this negative event does not represent a prediction error because they expect negative things to generally occur. However, this explanation would be inconsistent with the finding that resilience is associated with optimism—the expectation that positive events will occur in the future, despite significant evidence that a prediction error has occurred. This might represent a delusion if people had the belief that only great things can occur and that to maintain their well-being they have to deny this event occurred. Alternatively, if they believe that things tend to work out fine and that they can have good well-being despite these major life events, then these events would not represent well-being prediction errors per se. Evidence suggests that resilience is indeed associated with these types of optimistic beliefs (Segovia et al. 2012) that persist even in response to significant major life events (Leslie-Miller et al. 2021).

At the next level are those momentary positive and negative mood states that predict well-being. At this level, because those mood states reflect current situational and physiological conditions that need to be navigated to promote well-being, resilience ability should be associated with appropriately balancing updating belief states with maintaining belief states. For example, if an individual has a mild argument with a good friend, it might be more adaptive to maintain positive beliefs about this friend than to update the beliefs to be more negative. Doing so would likely increase the likelihood that this friend is able to contribute to one’s well-being in the future. However, if an individual finds out something nefarious about this friend (e.g., they run a dog-fighting ring), then updating one’s belief about them might be the best path to maintaining one’s future well-being. This hierarchical minimization of VFE (Figure 2) suggests that, whereas these higher-level mental models of well-being update slowly and reflect the association between resilience and well-being stability, the lower-level mental models are updated more flexibly in response to the environment, reflecting the association between resilience and flexible emotional and physiological responding to environmental challenges (Bonanno et al. 2004; Waugh et al. 2011).

### 4.1. Assessment of the Active Inference Model: Well-Being Beliefs and Forecasts

A key facet of maintaining stable positive well-being in the face of threats is the ability to make fairly accurate predictions about likely well-being changes. This process, known as affective forecasting, is central to resilience ability, such that individuals who form accurate models of how and when their well-being changes will be more likely to maintain positive well-being in the face of threats. Therefore, existing measures of affective forecasting, especially those that measure the forecasting of more global well-being changes (Dunn et al. 2003), might be a good performance measure for resilience ability. For example, scores from the Mayer–Salovey–Caruso Emotional Intelligence Test (MSCEIT; see Mayer et al. 2002), a performance measure of EI, but not self-reported measures of EI, significantly predict affective forecasting, with the management of emotion domain as the best predictor (Dunn et al. 2007). Thus, components of the allostatic active inference model may be captured by existing performance measures of EI.

Also critical to the allostatic active inference model is that VFE and the associated well-being beliefs are hierarchically situated, with stable well-being beliefs at the top and situationally flexible beliefs at the bottom. Reinforcement learning paradigms may be one promising type of measure for this VFE because they allow for the computation of moment-by-moment fluctuations in emotional responses to events as well as the overall stability of expected values of future decisions (Rutledge et al. 2014). Another promising measure might be to revise and extend the “strategic emotional intelligence” subscore of the MSCEIT to focus more on understanding well-being: beliefs about well-being (how stable it is, how easy it is to obtain and maintain, future expectancies of having well-being) and about the management of well-being (knowing when to adjust vs. maintain beliefs).

### 4.2. Assessment of Active Inference Model: Attentional Control and Updating

A potential foundational cognitive mechanism for how people can achieve this adaptive balance of well-being stability and emotional flexibility is attentional shifting. As our behavioral goals change, we must update the focus of attention or switch the prioritization of tasks held in working memory. While some environments require frequent updating, such as when navigating an unfamiliar environment, others require prolonged stable sustained selection, such as reading a book in a noisy coffee shop. These states of high and low switching readiness are referred to as cognitive flexibility and cognitive stability, respectively. Individuals possess the ability to harness previous experiences to learn to anticipate upcoming cognitive demands and adapt switching readiness accordingly, meaning that the cost associated with executing a shift of attention (Sali et al. 2015, 2020, 2022) or with executing a task switch (Chiu and Egner 2017; Dreisbach and Haider 2006) is smaller in contexts associated with a high likelihood of switching than in those with a low likelihood of switching. Although the relationship between individual differences in this metaflexibility of attentional control and in measures of well-being/emotional flexibility is not yet well understood, existing research has linked impairments in attentional control to trait anxiety (Eysenck et al. 2007). Thus, an important area for future study is whether metaflexibility in core processes like attention is associated with metaflexibility in processes associated with well-being maintenance.

The potential role of attention as an underlying cognitive mechanism in our model also provides a link to current models of EI. As noted above, one popular model suggests that EI is reflected in a hierarchical organization of four branches that range from basic information processing to more complex regulatory processes (Mayer et al. 2016). Recently, others have proposed emotional attention regulation as an additional ability associated with EI (Elfenbein et al. 2016; Elfenbein and MacCann 2017) and validated the Tuning in to and out of Nonverbal Cues of Emotion (TIONCE) as a measure of this ability (Elfenbein et al. 2016; Elfenbein and MacCann 2017). The TIONCE is a combination of auditory and visual Stroop tests in which participants must either direct selective attention to nonverbal emotion information (e.g., the vocal tone of a speaker’s voice) while ignoring the semantic content of the stimulus or ignore the nonverbal emotion information while attending to the semantic content. As noted above, an interesting question for future research is the degree to which metaflexibility of attention (e.g., regulating shift readiness across changing environmental context) is associated with resilience ability. The TIONCE offers one possible measure for studying when and how individuals direct selective attention to emotion cues and how directing attention away from emotion cues may in some circumstances aid in the stable maintenance of high well-being.

### 4.3. Assessment of Active Inference Model: Computational Modeling

Lastly, there have been nice computational models that reflect this hierarchical organization of stability and flexibility. One such model is the attractor model (Kuppens et al. 2010), which assesses how people’s affect tends to fluctuate over time (variability) and return to (or “be attracted to”) a “home base,” a relatively stable affective state. This affective home base is consistent with our notion of well-being maintenance, which is supported by findings that this home base is positive for those high in positive affect and life satisfaction (Kuppens et al. 2010). We would therefore expect it to be positive for those high in resilience ability as well. Lastly, the fluctuating affective states return to the affective home base with a certain “attractor strength,” which reflects the regulatory processes that we address in the next section.

## 5. Resilience Tools to Maintain Well-Being: Regulatory Flexibility

Whereas VFE is about belief and prediction updating, expected free energy (EFE) is about basing actions on predictions. Minimizing EFE involves maximizing the expected reward and/or information gained from a particular action (Smith et al. 2022). The batter did not swing because the expected reward from the predicted nonhittable pitch was to get on base and potentially score. Individuals with strong resilience ability should possess the capacity to select actions that maximize both reward and the information gained about their current environment in the service of maintaining well-being.

Maximizing reward in a given moment typically reflects the maximization of expected positive emotions and the minimization of expected negative emotions from a given action. This characterization is not as helpful when considering resilience ability, however, because although resilience tends to be associated with increased positive emotions (Waugh et al. 2011) even in response to threats to well-being (Fredrickson et al. 2003), it is not consistently associated with decreased negative emotions (Fredrickson et al. 2003; Waugh et al. 2011). Also, sometimes maximizing positive emotions and minimizing negative emotions might not be adaptive—as in the case of addiction and avoidance, respectively. More useful is to think of maximizing reward as enacting those actions that have the greatest likelihood of maintaining well-being. In this case, the “reward” is the maintenance of well-being itself. This allows for resilience ability to instead be associated with effective and adaptive affect regulation when confronted with a threat to well-being (Troy et al. 2023) that is tailored to the situation in such a way as to provide the greatest possible chance of leading to the maintenance of well-being.

This “strategy-situation” fit in coping is the current gold standard for understanding how different strategies can be effective in different types of situations (Park et al. 2001). For example, in high-control situations, strategies that focus on solving the problem tend to be more effective than those that only focus on changing one’s thoughts (O’Mara et al. 2011). Those high in resilience ability are able to successfully match the regulatory strategies to fit the situation (Bonanno and Burton 2013; Cheng et al. 2014). Resilient people exhibit adaptive affect regulation because they detect possible threats in their environment (as in our allostatic active inference model), select a candidate strategy from a repertoire of possible regulatory strategies and then maintain that strategy if effective or switch to another strategy if not (Bonanno and Burton 2013). Sometimes, these strategies might even promote negative emotions (Bonanno et al. 2004; Tamir et al. 2008; Waugh et al. 2011) as long as those negative emotions in the moment can serve to promote the maintenance of well-being in the long term (e.g., Westphal et al. 2010). For example, being angry and disappointed in your dog-fighting friend might improve your well-being over the long term by energizing your split from them.

The other aspect of minimizing EFE is maximizing information gain (Smith et al. 2022). This balance of reward and information gain suggests that if an individual with high resilience ability does not know which actions will promote well-being in a particular context, they will select the action that allows them to maximally learn about their environment. Once they have learned more about their environment, they can then select actions that maximally promote well-being maintenance. This information-gathering behavior is consistent with research showing that one of the most reliable predictors of resilience in children is their competence and self-efficacy (Masten 2001). Resilient children are able to collect information about their environment, process it deeply and then formulate solutions to problems (Masten and Coatsworth 1998). This environmental mastery then allows for resilient people to prevent or manage the stressor itself (problem-focused coping; Stratta et al. 2015) and/or understand the specific demands of the stressor in order to select the appropriate coping strategy (Bonanno and Burton 2013).

### Assessment of Regulatory Flexibility

We point readers to Bonanno and Burton (2013) for a comprehensive regulatory flexibility model and suggestions on how to assess each component. Our notion of “information-gathering” in our model is akin to their notion of “context sensitivity (evaluating demands and opportunities in the environment” and our notion of “maximizing expected rewards” is akin to their notion of “feedback (monitoring how well coping strategies are working and modifying as needed).” In addition, their model provides a nice process for how resilient people are able to balance these two sources of EFE. The “emotion management” module in the MSCEIT would also provide a good companion measure of general emotion regulation ability.

Note, however, that our notion of regulatory flexibility does not necessarily include specific regulatory strategies that have often been associated with well-being such as positive reappraisal (changing the one way thinks about a stressor in order to feel better about it; McRae and Mauss 2016) or positive distraction (taking time out from a stressor to do something pleasant; Waugh et al. 2020). These are not “resilience tools” per se because they are strategies that may or may not work given a particular situation, so they are just part of a toolbox that may also include other seemingly maladaptive (tending to be negatively associated with well-being) strategies like avoidance and suppression (Waugh et al. 2023). We contend that these “adaptive” sets of tools seem to be related to resilience because the situations they fit tend to be experienced more often than the situations in which they do not fit, and vice versa for the “maladaptive” sets of tools. Therefore, habitual use of these adaptive and maladaptive tools tends to predict good and poor outcomes, respectively. However, this conceptualization also leaves open the possibility that the “maladaptive” tools may be adaptive in some situations (e.g., denial at the beginning of a tragedy: Lazarus 1983) and that if someone experiences a particular set of situations more often than others, then the habitual “adaptiveness” of these tool sets may shift accordingly (e.g., threat-sensitivity in abused children: Thompson et al. 2014).

## 6. Resilience Tools to Maintain Well-Being: Positive Emotion as a Special Case

One of the most consistent findings in resilience research is that people who exhibit resilient outcomes tend to experience greater positive emotions in times of stress (Folkman 2008) such as responding to national tragedies (Fredrickson et al. 2003), coping with illness (Moskowitz 2003) and caregiving for ill people (Moskowitz et al. 1996). This prevalence of positive emotions has spurred models of resilience that argue that positive emotions and the positive appraisals that cause them are the primary mechanism behind resilience (Kalisch et al. 2014; Waugh and Koster 2015). Given their importance to resilience, positive emotions must fit into our resilience ability model, but how?

First, as we mentioned before, the shared mechanism of all forms of well-being is positive appraisal (Caprara et al. 2010; Waugh 2023). Hedonic well-being is a positive appraisal of one’s life and circumstances and eudaimonic well-being is a positive appraisal of one’s goal and meaning. Therefore, when we posit that resilience ability is “maintaining high well-being…” then we can substitute “high well-being” with “positive appraisals of one’s life, circumstances, etc.” Resilient people are particularly adept at making these global positive appraisals (Kalisch et al. 2014), which should translate to being adept at making situational positive appraisals that produce positive emotional states in the moment (Grandjean et al. 2008). Furthermore, if resilience ability is about maintaining those global positive appraisals despite threats to well-being, then we should expect that to translate to maintaining situational positive appraisals (and the associated positive emotions) during stressors, and that is indeed what researchers find (Fredrickson et al. 2003; Tugade and Fredrickson 2004).

Second, our model posits that one of the primary tools that resilient people use is monitoring well-being and one of the signals of having well-being is positive mood. The mood-as-information theory states that when making judgments about well-being-related aspects of our lives, people use their current mood as part of the basis of those judgments (Schwarz and Clore 2007). Researchers showed, for example, that people report higher well-being on days with good weather than on days with bad weather (Schwarz and Clore 1983) and that positive moods impact people’s evaluation of their meaning in life (King et al. 2006). This is not to say that global well-being is interchangeable with momentary moods (as noted in our section on well-being maintenance above), but that momentary moods and emotions serve as indices of well-being that motivate the actions required to maintain global well-being (Barrett 2017). For example, hanging out with friends may contribute to the maintenance of global well-being, but doing so does not require a full appreciation of its global well-being effects, just an appreciation that being with friends produces positive moods.

Third, in the allostatic active inference model described above, maintaining well-being is a predictive process, which means that resilience ability should be related to preparing for potential threats to well-being before they occur. Preparing for those threats requires gathering and maintaining the resources “in peacetime” needed to address them, and Fredrickson’s broaden-and-build model suggests that positive emotion is the primary mechanism of resource gathering and maintenance (Fredrickson 1998, 2001). When pursuing positive emotional experiences, people build social resources (like forming new friendships; Waugh and Fredrickson 2006), physical resources (better physiological health; Kok and Fredrickson 2010) and intellectual resources (like creativity; Isen et al. 1987). Furthermore, these resources built through the experience of positive emotions can be used to address future threats to well-being (Fredrickson 1998, 2001). For example, an individual may form a new friendship because being with this person is fun and comforting and then that friend may provide social support when the individual is undergoing a divorce.

### Assessment of Positive Emotions during Stress

Fortunately, there are many examples of how to assess positive emotions during stress. In a typical paradigm, participants undergo a stressor in the lab (such as an adapted version of the Trier Social Stress Test; Kirschbaum et al. 1993; Tugade and Fredrickson 2004) and report on their positive and negative emotions throughout. It has been consistently shown that resilient people are able to report higher positive emotions when recovering from stress (Fredrickson et al. 2003; Tugade and Fredrickson 2004) and that those higher positive emotions are related to their ability to make positive appraisals about the stressor (Kalisch et al. 2014; Tugade and Fredrickson 2004).

## 7. Conclusions

We have argued that, like EI, resilience is an ability and that ability is the maintenance of well-being despite threats to that well-being. As an ability, it is present in people before the threat to well-being occurs and is trait-like but can also be learned throughout life. The tools of resilience ability can be described with an allostatic version of an active inference model in which people high in resilience ability monitor well-being, minimize variational free energy by maintaining stable well-being beliefs while updating situational beliefs and minimize expected free energy by either prioritizing actions that are expected to lead to well-being maintenance or by gathering the information needed to discern which actions would lead to well-being maintenance. This model also accounts for the role that positive moods and emotions play in resilience as indices of well-being and potential well-being changes and producers of behaviors that promote well-being maintenance. This model should make at least three contributions to research on resilience and EI: (1) it advances some traditions in resilience that make a strong case for resilience as an ability that is present in varying levels across people and not just an outcome; (2) it provides a starting point for the assessment of resilience ability in terms of EI tools as well as other related tools, although future research is needed to fully flesh out the computational affect dynamics that can accurately capture it; and (3) it outlines one way in which high EI can predict resilience when coupled with the ability/goal to maintain well-being despite threats to that well-being.
